# Accouchement sur utérus cicatriciel dans les pays à faibles ressources: circuit de prise en charge et devenir materno-fœtal

**DOI:** 10.11604/pamj.2018.30.255.15678

**Published:** 2018-08-06

**Authors:** Valère Mve Koh, Henri Essome, Julius Dohbit Sama, Pascal Foumane, Bénédicte Mengue Ebah

**Affiliations:** 1 Faculté de Médecine et des Sciences Biomédicales, Université de Yaoundé I, Yaoundé, Cameroun; 2 Centre Hospitalier et Universitaire de Yaoundé, Yaoundé, Cameroun; 3 Faculté de Médecine et des Sciences Pharmaceutiques, Université de Douala, Cameroun; 4 Hôpital Gynéco et Pédiatrique de Yaoundé, Yaoundé, Cameroun

**Keywords:** Utérus cicatriciel, circuit de prise en charge, Yaoundé, Uterine scae, healthcare chain, Yaoundé

## Abstract

Le taux d'utérus cicatriciel, facteur de risque établi de la morbidité obstétricale augmente à travers le monde. Dans les pays en développement, les ruptures sur utérus non cicatriciel peuvent constituer 87,4% des cas. Sa prise en charge demeure ainsi une problématique de l'obstétrique moderne, notamment dans ces pays. L'objectif était de décrire le circuit de prise en charge et le devenir materno-fœtal des accouchées porteuses d'utérus cicatriciel dans trois hôpitaux universitaires de la ville de Yaoundé pour un état des lieux de la problématique de la prise en charge de cette morbidité grave dans les pays à faible ressources à l'aube des Objectifs pour le Développement Durable. Il s'agissait d'une étude descriptive, transversale avec collecte prospective des données, sur une période de six mois en 2014. Etaient inclues les accouchées consentantes, porteuse d'utérus cicatriciel, ayant accouché à un âge gestationnel supérieur ou égal à 28 semaines d'aménorrhée révolue. L'échantillonnage était consécutif et exhaustif. Chi carré a été le test utilisé statistiquement, P≤ 0,05 étant le seuil de fiabilité. Nous avons recruté 252 cas d'utérus cicatriciel, soit une fréquence de 8% (252/3145) pendant la période d'étude.30% avaient des CPN réalisées par un personnel inadéquat dans une structure sanitaire non appropriée,25% étaient référées avec complications pendant le travail après une première admission dans une formation sanitaire inadéquate, dont les 6 cas de ruptures utérines et 7 fœtus décédés avant l'admission.39% avaient une indication de césarienne/laparotomie dès l'admission, le taux d'accouchement par voie basse était de 23% , l'épreuve utérine était indiquée chez 30%, avec un taux de réussite de 76,3% .L'accouchement par voie basse était corrélé à la parité, les antécédents d'accouchement par voie basse , la macrosomie fœtale et inversement corrélé au nombre de cicatrices .Le nombre de décès maternel était nul et l'accouchement par césarienne était corrélé aux morbidités materno-fœtales. La mauvaise qualité des CPN et de la prise en charge de l'accouchement demeurent les principaux déterminants de la problématique de l'accouchement sur utérus cicatriciel dans notre milieu. La mise en place d'un système facilitant l'accessibilité des femmes enceintes porteuses d'utérus cicatriciel aux prestataires/formations sanitaires adéquats permettrait d'améliorer le pronostic des accouchées porteuses d'utérus cicatriciel.

## Abstract (en)

The rate of uterine scars, an established risk factor for obstetric morbidity, is increasing worldwide. In developing countries, spontaneous uterine ruptures may constitute 87.4% of cases. Tratment is a problem in modern obstetrics, in particular in these countries. This study aims to describe healthcare chain and materno-fetal follow-up of post-partum women with uterine scar in three university hospitals in the city of Yaoundé in order to highlight morbidity management problems in low-resource countries at the dawn of sustainable development goals. We conducted a cross-sectional descriptive study based on the collection of prospective data over a period of six months in 2014. The study included all consenting post-partum women with uterine scar, having given birth to a gestational at a gestational age greater than or equal to a total of 28 weeks of amenorrhea. The sampling was consecutive and exhaustive. Chi square test statistic was applied in all research areas, with a reliability threshold of p≤ 0.05. Data on 252 women with uterine scars, reflecting a rate of 8% (252/3145), were collected during the study period. Prenatal consultations were performed by inadequate staff in an inadequate sanitary structure in 30% of cases. Women were referred due to delivery complications after first admission to an inadequate sanitary structure in 25% of cases (6 uterine ruptures and 7 dead fetus before admission). There was indication for cesarean section/laparotomy on admission in 39% of cases; the rate of vaginal delivery was of 23%; there was indication for trial of scar in 30% of cases, with a success rate of 76.3%. Vaginal delivery was related to parity, a history of vaginal delivery, fetal macrosomia and was inversely related to the number of scars. Maternal mortality was zero and cesarean section was related to materno-fetal morbidity. The poor quality of prenatal consultations and the management of delivery are the main determinants of problems during vaginal birth after cesarean section in our environment. The establishment of a system facilitating access to skilled health care practitioners/adequate health care facilities for pregnant women with uterine scar would improve the prognosis of post-partum women with uterine scar.

## Introduction

La maternité sans risque est un des défis majeurs de la santé materno-fœtale et néonatale. Le taux de césarienne augmente à travers le monde, avec comme conséquence une fréquence en croissance des utérus cicatriciels [1], une des principales causes de morbidité telle que de la rupture utérine, entre autres [2]. Dans les pays en développement, les ruptures sur utérus non cicatriciel peuvent constituer 87,4% des cas de rupture [3], alors qu'elles sont rares en Occident sur utérus non cicatriciel. La problématique du pronostic materno-fœtal des utérus cicatriciels dans notre milieu demeure actuelle. L'objectif était de décrire le circuit de prise en charge et le devenir materno-fœtal des accouchées porteuses d'utérus cicatriciel dans trois hôpitaux universitaires de la ville de Yaoundé, afin de faire l'état des lieux de la problématique de la prise en charge de cette morbidité grave dans les pays à faible ressources, à l'aube des Objectifs pour le Développement Durable(ODD).

## Méthodes

Il s'agissait d'une étude descriptive, transversale avec collecte prospective des données, dans trois hôpitaux universitaires de la ville de Yaoundé, à savoir le Centre Hospitalier et Universitaire (CHUY), l'Hôpital Central (HCY) et l'Hôpital Gynéco-Obstétrique et Pédiatrique (HGOPY), sur une période de six mois, du 1^er^Janvier au 1^er^Juillet 2014. Etait considéré comme cicatriciel, tout utérus porteur d'une ou plusieurs cicatrices myométriales, du corps ou de l'isthme. La population d'étude était constituée d'accouchées porteuses d'un utérus cicatriciel, dans les maternités de ces trois hôpitaux. Etaient inclues les accouchées consentantes, porteuses d'utérus cicatriciel, ayant accouché à un âge gestationnel supérieur ou égal à 28 semaines d'aménorrhée révolue, calculée à partir de la date des dernières règles. Les accouchées non consentantes, ou avec cicatrice(s) intéressant uniquement la séreuse utérine étaient exclues. Le consentement était éclairé, après la clairance du comité d'éthique de l'université de Douala. Etait considéré comme prestataire de santé adéquat pour le suivi de grossesse de ces utérus cicatriciels, les gynécologues obstétriciens, les médecins généralistes et les sage femmes. Les formations sanitaires (FS) adéquates pour la prise en charge de l'accouchement désignaient celles qui avaient un plateau technique et des ressources humaines qualifiées (gynécologues /obstétricien), disponibles 24h/24 pour la prise en charge sans délai, de toutes les urgences obstétricales sans nécessité de référence. L'échantillonnage était consécutif et exhaustif. Le traitement et l'analyse des donnés se sont faites grâce au logiciel Sphinx millenium 4.5 (hilaire) et Microsoft office Excel 2013. Le χ^2^ a été le test utilisé pour les analyses statistiques, P≤ 0,05 étant le seuil de fiabilité.

## Résultats

Nous avons recruté 252 cas d'utérus cicatriciel, soit respectivement 48 au Centre Hospitalier et Universitaire, 89 à l'Hôpital Central et 115 à l'Hôpital Gynéco et Pédiatrique, trois hôpitaux universitaires de la ville de Yaoundé. 2888 accouchements ont eu lieu pendant la période d'étude, soit une fréquence de 8% (252/2888). Les âges des accouchées variaient de 19 à 42 ans, pour une moyenne de 30,7 ans. Plus de la moitié était mariée (55%), 84,5% avaient au moins un niveau d'instruction secondaire ou universitaire. Au moment de l'admission, la (ou les) cicatrice(s) antérieure(s) était(nt) une césarienne chez 98,4%, près de la moitie soit 6,7% avaient réalisé les consultations prénatales (CPN) dans des formations sanitaires inadéquates, dont un tiers (30,1%) dans des Centres de Santé Intégrés, niveau initial de la pyramide sanitaire. Concernant le prestataire des CPN, elles étaient réalisées par un prestataire de santé non approprié chez 31,3% et 18,7% n'avaient pas réalisé le minimum de quatre CPN requis alors (Tableau 1). Vingt-cinq pour cent avaient été référées/évacuées au moment de l'accouchement après une première admission dans une formation sanitaire non adéquate pour la prise en charge des utérus cicatriciels en travail, dont six ruptures utérines, après déclenchement de travail dans des Centre de Santés Intégrés, avec une anomalie ou absence de bruits du cœur fœtal chez un dixième (11,5%), une indication dès admission de césarienne d'urgence ou laparotomie exploratrice d'emblée chez 39%,sans aucun décès maternel (Tableau 2). L'antécédent d'accouchement par voie basse avant ou après la césarienne augmentait le nombre d'accouchement par voie basse (P<0,05 Tableau 3) et l'utérus multi-cicatriciel favorisait la voie haute, mais deux cas d'accouchées porteuses respectivement d'utérus bi cicatriciel et tri cicatriciel arrivées à dilatation complète, avaient accouché par ventouse obstétricale. (P= 0,05) (Tableau 4). Trente-trois pour cent des 15 macrosomes étaient nés par voie basse et vivants, et la proportion de l'accouchement par voie basse par intervalle de poids fœtal dans la série augmentait avec le poids fœtal (P≤ 0,05) (Tableau 5). Seul deux cas sur les 58 accouchées porteuses d'utérus cicatriciel ayant accouché par voie basse avaient un score d'Apgar à la cinquième minute inférieure à sept, les 12 autres étant nés par césarienne (P≤ 0,05) (Tableau 6), 12% des cas d'accouchement par voie basse présentaient des complications tels que la prématurité (2), la SFA (1), l'ANN (2), INN (1) et le décès périnatal, contre 16,5% dans le groupe accouchement par césarienne (Tableau 7).

**Tableau 1:** Récapitulatif des variables cliniques avant admission

Modalités	Effectifs	Fréquences (%)
**Chirurgies antérieures**		
Césarienne	244	98,4
Myomectomie	8	1,6
**Formation sanitaire des CPN**		
Centre de Santé Intégré*	76	30,1
Clinique Privée*	33	13,1
Hôpital de District*	9	3,5
HCY**	30	11,9
HGOPY**	76	30,1
CHUY**	28	11,1
**Prestataire des CPN**		
Gynécologues/Obstétriciens	170	67,4
IDE/Infirmier Accoucheur breveté***	79	31,3
Médecin généraliste	3	1,1
**Nombre de CPN avant admission/accouchement**		
< 4	47	18,7
≥ 4	205	82,3

* Formation sanitaire inadéquate

** Formation sanitaire adéquate

*** Prestataire non approprié

**Tableau 2:** Récapitulatif de variables cliniques pendant et après admission

Modalités	Effectifs	Fréquences (%)
**Mode d’admission (p≤0,05)**		
Evacuée/Référée des FS non adéquates	63	25
Admise en 1^ère^ Intention dans les hôpitaux adéquats	189	75
**Prise en compte de la documentation de la cicatrice**		
Oui	0	0
Non	252	100
**Rupture utérine à L’admission (post déclenchement de travail dans FS non adéquate)**		
Oui	6	2,4
Non	246	97,6
**déclenchement de travail dans FS non adéquate) Etat fœtal à L’admission (P≤ 0,05)**		
BDCF absents ou<120	29	11,5
ou>160/mn		
BDCF [120-160]	223	88,5
**Décision à l’admission**		
Césarienne d’urgence /Laparotomie exploratrice d’emblée**	98	38,9
Césarienne élective	70	30,9
Epreuve cicatrice puis Césarienne	18	7,1
Epreuve cicatrice puis AVB	58	23,1
**Décès maternel**		
Oui	0	0
Non	252	100

** Dont les six (6) ruptures utérines

FS= Formation Sanitaire

**Tableau 3:** Antécédent d’accouchement par voie basse avant ou après la césarienne et mode d’accouchement

Nombre de cicatrices	AVB	Césarienne	
Nombre de cicatrices	**Effectifs (%)**	**Effectifs (%)**	**Total**
Uni cicatriciel	56(29,5)	134(70,5)	190
Bi cicatriciel	1(1,8)	54(99,2)	55
Tri cicatriciel	1(16,6)	5(83,4)	6
Quadri cicatriciel	0	1	1
Total	58	194	252

**Tableau 4:** Nombre de cicatrices utérines et mode d’accouchement

Nombre de cicatrices	AVB	CESARIENNE	
Nombre de cicatrices	Effectifs (%)	Effectifs (%)	Total
Uni cicatriciel	56 (29,5)	134 (70,5)	190
Bi cicatriciel	1 (1,8)	54 (99,2)	55
Tri cicatriciel	1 (16,6)	5 (83,4)	6
Quadri cicatriciel	0	1	1
Total	58	194	252

**Tableau 5:** Poids de naissance et mode d’accouchement

Poids nouveau-né (g)	AVB	Césarienne	
Poids nouveau-né (g)	Effectifs(%)	Effectifs(%)	Total
<2500	2 (10)	18 (90)	20
2500-3500	35 (21,7)	126 (78,3)	161
3500-4000	16 (28,6)	40 (71,4)	56
≤4000	5 (33,3)	10 (66,7)	15
Total	58	194	252

**Tableau 6:** Score d’Apgar selon le mode d’accouchement

Score Apgar 5^ème^mn	AVB	Césarienne	
Score Apgar 5^ème^mn	Effectifs (%)	Effectifs (%)	Total
0	1(14,3)	6(85,7)	7
[1-7[	1(14,3)	6(85,7)	7
≥7	56(23,8)	179(76,2)	235
Total	58	194	252

**Tableau 7:** Complications périnatales selon le mode d’accouchement

Complications périnatales	AVB	Césarienne	
Complications périnatales	Effectifs (%)	Effectifs (%)	Total
ANN	2(20)	8(80)	10
Prématurité	2(25)	6(75)	8
Décès prénatal	1(7,6)	12(92,4)	13
INN	1(50)	1(50)	2
SFA	1(33,3)	2(66,7)	3
Détresse respiratoire	0	3(100)	3
Aucune	51(24)	162(76)	213
Total	58	194	252

**ANN:** asphyxie néonatale; **INN**: infection néonatale (via CRP); **SFA**: souffrance fœtale aigue

## Discussion

La fréquence d'utérus cicatriciel chez les accouchées était de 8%, comme la moyenne de 8,8% retrouvée dans une série multicentrique africaine concernant 83 439 accouchements dans 131 formations sanitaires [4]. En France métropolitaine, elle a augmenté de 8 à 11,5% entre 1995 et 2010 [2], et variait de 2,3 à 27,3% en Afrique de l'Est, pour un taux moyen de 13,4% [4]. Elle varie de 2,4 à 14% dans la littérature récente au sud du Sahara [5, 6]. La prévalence des grossesses sur utérus cicatriciel varie d'une structure sanitaire à une autre quel que soit le pays et est probablement liée au taux de césarienne. Dans notre série en effet, l'antécédent de césarienne représentait 98,4% des cicatrices utérines, et le taux de césarienne global, de 19,84% pendant la période d'étude. Le suivi prénatal des femmes porteuses d'utérus cicatriciel a mis en évidence la problématique de la qualité de celui-ci. En effet la consultation prénatale recentrée est un pilier établi de la lutte contre la morbidité et la mortalité materno-fœtale [7], mais près de la moitié des femmes enceintes porteuses d'utérus cicatriciel de cette série avaient réalisé toutes les consultations prénatales dans des formations sanitaires sans médecin ni sage-femme, dont plus d'un tiers dans des formations sanitaires ne devant fournir que des soins obstétricaux et néonataux de base, structurellement non recommandées pour la prise en charge ce type de grossesse. Un cinquième n'avaient même pas réalisé le minimum de quatre visites prénatales comme recommandé alors par l'OMS.

La consultation prénatale recentrée permet en effet de détecter et de traiter précocement les morbidités materno-fœtales, la promotion de la santé, volet au cours duquel les complications associées à l'utérus cicatriciel auraient pu être évoquées, et le plan d'accouchement qui indique le lieu le plus approprié de celui-ci, le prestataire compétent devant prendre en charge l'accouchement sur utérus cicatriciel etc.[8], mais un quart n'avaient pas choisi, au moment du travail, en première intention, une formation sanitaire fournissant des soins obstétricaux et néonataux complets, et avaient été référées pour complications au cours du travail, amplifiant l'effet des retards à la prise en charge, facteur confirmé de morbi-mortalité en santé de la reproduction selon le modèle des trois retards [9], avec pour conséquences, six admises avec des ruptures utérines, pour déclenchement de travail par prestataire non indique dans des formations sanitaires sans bloc opératoire, dont un cas ayant nécessité une hystérectomie subtotale d'hémostase. Mbassi et al avaient montré que la référence multipliait les complications materno-fœtales de deux à quatre fois, tout type de grossesse confondu [10]. Ce suivi non optimal des femmes enceintes porteuses d'utérus cicatriciel est fréquent dans la littérature africaine [11-13]. La consultation prénatale de qualité est un des trois piliers de la lutte contre la morbi-mortalité maternelle et néonatale, et son importance n'est plus à démontrer [7]. Elles ont été ainsi le principal levier modifié dans les recommandations de l'OMS de novembre 2016, avec désormais huit visites minimales recommandées, dans le cadre des ODD [14]. Elle est obligatoire par exemple en France, toute grossesse confondue, et est prise en charge par la Sécurité Sociale, permettant un suivi prénatal de bonne qualité. La Haute Autorité de Santé a même établi les types de suivi et structures sanitaires recommandées pour l'accouchement en fonction des situations à risque [15]. Nous n'avons pas retrouvé de telles recommandations africaines concernant l'utérus cicatriciel, au cours de ce travail.

L'accessibilité financière est une des problématiques identifiées. Eloundou et al ont récemment montré que les fonctionnaires salariés et les commerçants représentaient 92% des femmes enceintes ayant pu réaliser le minimum des quatre visites recommandées, alors qu'aucune des ménagères n'avaient pu le faire [16]. Cette étude a été pourtant menée dans une zone couverte par la politique de chèque-santé, qui consiste en une contribution financière unique de 13 dollars US pour la prise en charge totale de la grossesse et de l'accouchement. Les accouchées sans activités génératrice de ressources représentaient 70,6% de notre échantillon. Toutefois, le fait que cette modique contribution n'ait pas amélioré l'accessibilité aux CPN, dans cette zone islamisée, montre l'effet d'autres facteurs associés. La littérature récente a identifié des facteurs d'ordre socioculturel, (religion, niveau d'instruction, habitation en zone rurale…), cliniques (la parité…) économiques (distance pour atteindre la formation sanitaire de prise en charge etc. [17]. Aux Etats-Unis, les facteurs associés au début tardif de CPN par exemple étaient l'adolescence, la grande multiparité, le faible niveau d'éducation, les situations économiques précaires [18]. La qualité des consultations prénatales, lorsqu'elles sont accessibles, est une autre problématique. Dans une série au Sénégal, les raisons de mauvaise qualité des CPN, selon le diagramme de pareto étaient entre autres le retard des femmes enceintes à la première consultation, l'insuffisance de personnel qualifié, le manque de motivation, l'insuffisance des recyclages, le remplacement des personnels formés, l'insuffisance en matériel technique, l'ignorance des bénéficiaires… [19]. Notre étude n'a pas investigué cette problématique. Concernant le mode d'accouchement, notre fréquence d'épreuve utérine, soit 30% était conforme à celle retrouvée dans la littérature, avec un taux de réussite de 76,3%, soit une fréquence d'accouchement par voie basse après césarienne de 23%, malgré la référence d'un quart des cas. En effet, l'épreuve utérine est indiquée dans 27,8 à 88,2% de cas, avec un taux de réussite qui varie de 45 à 92,2% [20]. Nos données sont inférieures à la moyenne et s'expliquerait probablement par la proportion des utérus multi cicatriciels dans notre série, soit 24%. Celles-ci demeurent une indication de la voie haute dans notre milieu. Toutefois, Tahsen et al ont retrouvé dans une méta analyse un taux d'accouchement par voie basse de 71,1% avec une fréquence de rupture utérine relativement faible (1,36%) en cas d'utérus bi cicatriciel [21]. Le même constat a été fait par une méta analyse récente qui estimait que l'utérus bi cicatriciel, ou associé à une grossesse gémellaire et l'absence d'indication récurrente n'étaient plus des contre-indications de la voie basse [22], l'appréciation de la qualité de la cicatrice par imagerie médicale doit être dans ces cas un prérequis [23]. Nous avons retrouvé un cas d'accouchement instrumental chez utérus tri cicatriciel référée, admise au second stade du travail, et aucune documentation concernant la ou les cicatrices antérieures n'était systématiquement recherchée ni prise en compte.

Le risque d'accouchement par césarienne peut être multiplié par 14 en cas d'utérus cicatriciel [24] et par 27,4 par la référence [25] .Dans notre série, une césarienne/laparotomie exploratrice était indiquée dès l'admission chez 39% des cas, alors que seules 25% avaient été référées, mettant probablement en évidence les premiers et deuxièmes retards du modèle dit des trois retards, y compris chez celles qui avaient choisies les hôpitaux universitaires en première intention au moment de l'accouchement, dont probablement des cas non recensés de césarienne élective arrivés en travail. Un plan d'accouchement de qualité aurait pu éviter ce retard évoqué lors de la CPN recentrée. Selon l'OMS en effet, la plupart des morbidités maternelles résultent du retard dans la décision de rechercher des soins, retard dans la réalisation des soins et le retard dans l'administration des soins de qualité [26]. Dans une cohorte concernant 2635 accouchement sur utérus cicatriciel, l'accouchement par voie basse était favorisé par l'âge maternel < 30ans, l'antécédent d'accouchement par voie basse avant ou après la césarienne [27], l'absence d'indication récurrente entre autres [28], le travail spontané, l'obésité, la race [29]. Cette étude a fait le même constat, l'absence d'antécédent d'accouchement par voie basse avant ou après césarienne multipliant par deux le nombre d'accouchement par voie basse (P< 0,005). Le mode d'accouchement de l'utérus uni cicatriciel en présence de macrosomie demeure une problématique de l'obstétrique moderne. Les nouveau-nés au poids ≥ 4000 g représentaient 6% de l'effectif, avec une proportion d'accouchement par voie basse de 33%, supérieure à celle des poids fœtaux < 4000 gr (22,3%) (P< 0,05), même si cette étude présente comme limite, un biais de sélection lié à la période relativement courte de recrutement, et n'a pas évoqué le poids maximum des macrosomes. Nous n'avons pas retrouvé dans la littérature une augmentation du taux d'accouchement par voie basse proportionnelle au poids fœtal en contexte d'utérus cicatriciel. Abdoulfalah et *al*ont autorisé 83,7% d'épreuve utérine chez 297 cas de macrosomie sur utérus cicatriciel, avec 63,3% d'accouchement par voie basse, certes compliqués de 12 ruptures ou déhiscence utérine, les auteurs estimant que ces résultats étaient statistiquement similaires à ceux observés dans le groupe césarienne élective [30]. Zelop et al estimaient que la macrosomie fœtale n'était pas une contre-indication de l'épreuve utérine mémé si au delà de 4250 g une prudence était recommandée [31]. Al Zirqi et *al* par contre ont montré que la macrosomie, en contexte d'utérus non cicatriciel déjà, augmentait le risque de rupture d'un ratio qui variait de 1,3 à 3,1 [32], tout comme dans la méta analyse de Landon où le poids fœtal< 4000g était un facteur favorisant le succès de l'accouchement par voie basse après césarienne [29].

Ces constats contradictoires y compris le nôtre, mettent-ils en évidence le rôle d'autres déterminants anthropométriques du macrosome? Dans notre série, nous avons eu une fréquence globale de 2,4% de ruptures utérines, mais aucun cas après l'admission. Elle était cinq fois plus élevée que la moyenne d'une enquête à travers 29 pays et deux fois et demi celle retrouvée dans les pays similaires [33]. Dans une série norvégienne sur quatre décennies, la cicatrice utérine pouvait augmenter de 74 fois la fréquence des ruptures utérines, et était corrélée entre autres au déclenchement ou à la stimulation du travail [34]. L'hypothèse, non investiguée dans cette série, d'un faible taux de déclenchement et de stimulation de travail, en contexte d'utérus cicatriciel, observé empiriquement dans notre milieu , est probable, tout comme l'est celle de la contribution de la qualité de la prise en charge dans les hôpitaux d'études, à savoir, des hôpitaux universitaires, avec personnel qualifié et plateau technique disponible 24h/24. L'accouchement assisté par personnel compétent est un pilier de la lutte contre la morbidité/mortalité maternelle, même si un biais de sélection dû à la durée de l'étude soit six mois peut relativiser ce constat. Concernant les complications fœtales, les données étaient conformes à la littérature, à savoir, une exacerbation des complications dans le groupe césarienne notamment d'urgence, versus voie basse [35]. En effet, l'épreuve utérine réussie avait moins de complications fœtales que la césarienne, avec une contribution probable des morbidités ayant conduit à son indication. Certains auteurs, estiment en effet qu'il y'a plus de morbidité dans le groupe échec de la voie basse [36]. Dans cette série 1/3 avait été référé mais seule 2,4% des 246 fœtus vivant à l'admission sont décédés au cours de la prise en charge mettant en exergue l'importance de l'accouchement assisté par personnel qualifié

## Conclusion

La prise en charge de l'accouchement sur utérus cicatriciel dans notre milieu est encore caractérisée par un suivi de grossesse inapproprié, un retard à la prise en charge de l'accouchement par personnel adéquat, une référence au stade des complications. La macrosomie pourrait ne pas contre-indiquer l'accouchement par voie basse. L'accouchement assisté par personnel qualifié dans une formation sanitaire adéquate réduit la mortalité maternelle et les morbidités périnatales, et devrait être recommandé. L'éducation/sensibilisation des femmes enceintes porteuses d'utérus cicatriciel et l'amélioration de leur accessibilité, dans les formations sanitaires de prise en charge définitives peuvent améliorer le pronostic maternel et périnatal.

### Etat des connaissances actuelles sur le sujet

- L'antécédent d'accouchement par voie basse avant ou après la cicatrice comme facteur de pronostic positif pour l'accouchement par voie basse après césarienne;
- L'accouchement assiste par personnel qualifie comme facteur prédictif du bon devenir materno-fœtal;
- Le risque majoré de rupture utérine si accouchement par voie basse après césarienne.

### Contribution de notre étude à la connaissance

- L'évidence de la possibilité d'accouchement par voie basse de macrosome après une césarienne en contexte de faibles ressources;
- La moindre importance de la documentation de la cicatrice antérieure en contexte d'accouchement sur utérus cicatriciel;
- La fréquence d'accouchement par voie basse du macrosome proportionnel au poids fœtal en contexte d'utérus cicatriciel dans un environnement a faible ressources.

## Conflits d'intérêts

Les auteurs ne déclarent aucun conflit d'intérêt.
